# Towards equal representation - A bibliometric analysis of authorships in Laboratory Medicine and Clinical Chemistry from the United States, Canada, and Europe (2005–2022)

**DOI:** 10.1016/j.heliyon.2024.e31411

**Published:** 2024-05-16

**Authors:** Annika Meyer, Thomas Streichert

**Affiliations:** Institute of Clinical Chemistry, Faculty of Medicine and University Hospital, University Hospital Cologne, Kerpener Str. 62, 50937 Cologne, Germany

**Keywords:** Gender, Authorship, Representation, Equality, Clinical chemistry, Laboratory medicine, Bibliometric

## Abstract

**Objectives:**

Although diversity has been demonstrated to benefit research groups, women remain underrepresented in most scientific disciplines, including Laboratory Medicine and Clinical Chemistry. In order to promote diversity and equality in scientific communities, understanding the gender distribution of authorship is crucial.

**Methods:**

This study included a total of 30,268 Web of Science-listed Clinical Chemistry and Laboratory Medicine publications from the United States of America, Canada, and the member countries of the European Federation of Clinical Chemistry and Laboratory Medicine from 2005 to 2022. In addition to the publication productivity of female and male authors over time, gender-specific publication characteristics and country-specific gender distributions of authorships were examined.

**Results:**

Overall, publications with female first authors increased by 49 % between 2005 and 2022, averaging 42 % female first authors. Eastern Europe (60 %) and Southern Europe (51 %) had particularly high proportions of female first authors. While female last authorship was the most predictive of female first authorship, with an odds ratio of 2.01 (95 % CI: 1.91–2.12, p < 0.001), only 27 % of last authors were female. Moreover, citation rate was not predictive of female first or last authorship.

**Conclusion:**

Authorship in Clinical Chemistry and Laboratory Medicine is moving towards gender parity. This trend is more pronounced for first authors than for last authors. Further research into the citations of female authors in this discipline could be a starting point for increasing the visibility of women researchers in science. Moreover, geographical differences may provide opportunities for future research on gender parity across disciplines.

## 1 Introduction

Publication of scientific articles is an important step in the research process to present results to the scientific community, thereby encouraging discussion and promoting development. In this context, authorship allows accountability, rewards scholarly achievement, and facilitates career advancement [1]. It can shape the methodological development and thematic orientation of their respective disciplines [2]. However, authorship also reflects the distribution of power and gender within scientific communities [3].

Embracing diversity in authorship not only increases the productivity of research groups [4,5], but also broadens research interests and priorities [2,[6], [7], [8]]. Yet, female authors remain a minority in research [9,10], despite representing a near majority of the world's population [11] and 39–42 % of students in science, technology, engineering and mathematics (STEM) as well as 69–73 % of medical students in Europe and North America [12].

The literature attributes this mismatch to inequality in the workplace [13], higher rates of rejection in the peer-review process [14] and less authorship credits for equal scientific contribution [13,15]. Since female authors face country and discipline-specific challenges, gender distribution varies [16], leaving either role models or contrary examples.

As Laboratory Medicine and Clinical Chemistry attracts researchers with medical, pharmacological and chemical backgrounds [17], its authorship distribution not only yields insights into its field but also interdisciplinary collaboration within a unique work environment. Therefore, understanding the gender distribution of authorship is crucial in promoting diversity and equality in scientific fields.

Thus, the objective of this study is to investigate the gender distribution of authorship in Laboratory Medicine and Clinical Chemistry from Canada, the United States and Europe between 2005 and 2022.

## 2 Methodology

1. Initial Data Collection

In this bibliometric study, we examined the gender distribution of authorship in Laboratory Medicine and Clinical Chemistry using 30,268 publications. We chose Web of Science for data collection since it is particularly suitable for bibliometric studies due to its extensive library and detailed metadata documentation [18,19]. In constructing our search query, we identified journals associated with the European Federation of Clinical Chemistry and Laboratory Medicine, the United States and Canada by name and ISSN. In addition, we supplemented our search with the strategy of Campos et al. [20] and Clinical Chemistry synonyms [21] using the Web of Science topic field tag.

2. Exclusion and Inclusion Criteria

**Fig. 1:**
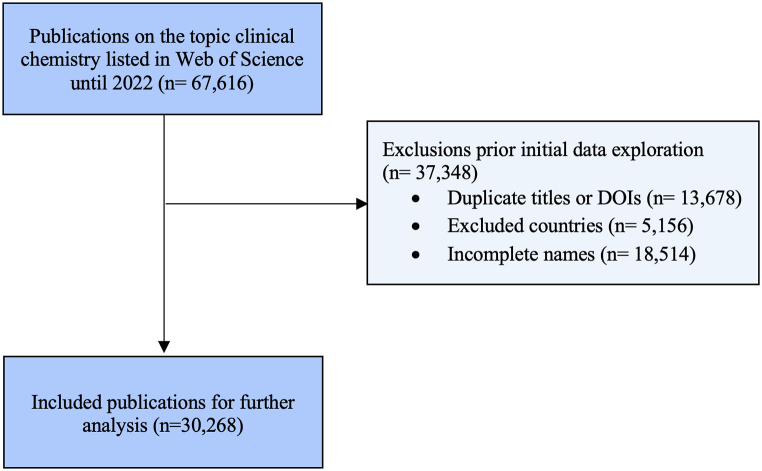
Flowchart illustrating the selection process of Clinical Chemistry publications through the application of inclusion and exclusion criteria, 2005–2022.

Exclusion criteria were defined as duplicate titles or DOIs and incomplete authors’ names. To refine our dataset and improve the accuracy of the gender assignment algorithm, we then excluded publications with first authors not affiliated in the United States, Canada or member countries of the European Federation of Clinical Chemistry and Laboratory Medicine. This allowed us to exclude 37,348 of the 67,616 records, leaving 30,268 records for final analysis (Fig. 1).

3. Gender Assignment

In most scientific disciplines, the order of authorship reflects the proportion of work. The last authorship is usually awarded to the principal investigator, whereas the first author has usually made the greatest contribution to the publication [22,23]. In recognition of the role these positions typically play in the scientific community, we therefore decided to identify the gender of the first and last authors in our study.

To examine the gender distribution of authors, we followed the existing literature by using a first name-based approach [16]. Although we recognise that gender is a complex construct that cannot be fully captured by a binary classification, we consider this method to be a compromise for detecting trends in gender distribution among authors.

Following the recommendation of Sebo et al. [24], we used a mixed methods approach with Wiki-Gendersort [25] and genderizeR (genderize.io API) [26] to assign gender to name.

4. Statistical Analysis

The R language was used for the statistical analyses (see Supplemental 1 for details of the packages used). Descriptive statistics presented categorical variables as medians and interquartile ranges. Continuous variables were shown as means and standard deviations. We measured publication productivity by the Compound Annual Growth Rate from 2006 to 2021. Given the potential for error in tests of normal distribution with large sample sizes, we relied on QQ plots for the graphical analysis of normal distribution. Non-normally distributed continuous variables were compared using the Kruskal-Wallis test, while normally distributed variables were analyzed using one-way ANOVA. Chi-squared test was used for categorical variables. Statistical significance was defined as less than 0.05. After scaling continuous variables, univariate and multivariate regression were used to supplement descriptive statistics.

The role of the “Deutsche Forschungsgemeinschaft” (German Research Foundation) was limited to contributing the publication fee for the article and was not involved in study design, data collection, analysis or interpretation, or manuscript preparation.

## 3 Results

1. Gender Distribution

**Fig. 2:**
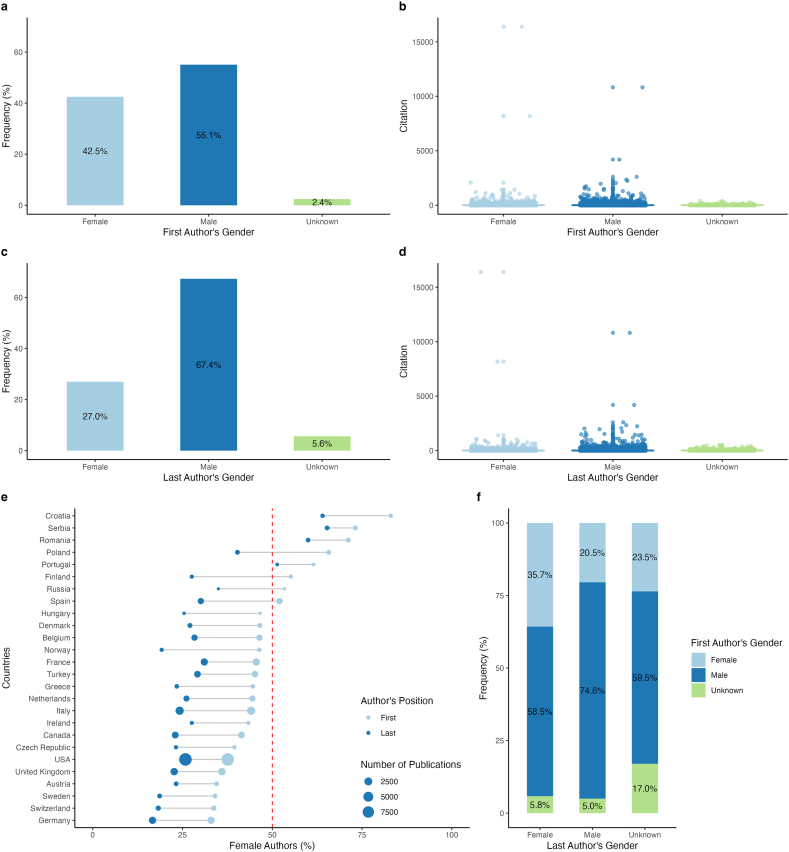
**Gender-based authorship distribution in Clinical Chemistry and Laboratory Medicine, 2005**–**2022.** Bar graphs (Fig. 2a–c, Fig. 2f) illustrate publications by gender and authorship position. Publications citations per gender are illustrated by box plots (Fig. 2b and d). Relative frequency of female authorship per first authors' affiliated country with more than 100 publications is highlighted by the lollipop graph (Fig. 2e).

The analysis of 30,268 Clinical Chemistry and Laboratory Medicine publications revealed almost gender parity among first authors with 42.5 % female (12,861/30,268), 55.1 % male (16,6721/30,268) and 2.4 % unknown first authors (7351/30,268; q < 0.001) (Fig. 2a), whereas last authorship was not balanced (men = 20,389/30,268 (67 %), women = 8175/30,268 (27 %), unknown = 1704/30,268 (5.6 %); q < 0.001) (Fig. 2d). Female first and last authors collaborated 56.2 % of the time, whereas only 36.9 % of male last authors co-authored with female first authors (q < 0.001) (Fig. 2g). The odds ratio of 2.01 (95 % CI: 1.91–2.12, p < 0.001) also reflected this significant association between female first and last authorship (Supplemental Fig. 1).

**Fig. 3:**
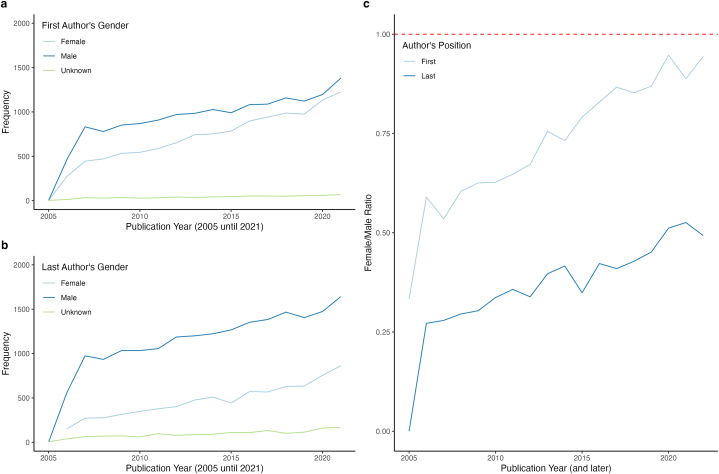
**Gender-based authorship distribution in Clinical Chemistry and Laboratory Medicine over time.**Fig. 3a displays the female to male authorship ratio for first (light blue) and last authorship (dark blue) over time, with the red line indicating a 1:1 gender ratio. The gender-specific frequency of first authorships is shown in Fig. 3b and last authorships in Fig. 3c.

Over time, however, the difference between male and female authors narrowed as women's publication productivity exceeded that of male authors. Specifically, between 2006 and 2021, female first authors had a productivity rate of 10.53 %, while female last authors reached 12.27 %, compared to 7.55 % and 7.45 % respectively for male authors (Fig. 3a and b), leading to an increase in the ratio of women to men over time (Fig. 3c).

## 4 Publication and author characteristics

Moreover, a comparison of publications by female and male first authors revealed several differences, including a significantly lesser citation frequency of female authored publications (Table 1). Nonetheless, citationrate was not a significant predictor for female first or last authorship (Supplemental Fig. 1). Factors that were predictive of higher citation rates included a lower number of authors per publication, an earlier year of publication, a higher usage count, the absence of author keywords, document type, and the affiliated country of first authors (Supplemental Table 1). Similarly, neither usage count since 2013 nor impact factors were predictive of female last authorship (Supplemental Fig. 1).

**Table 1:** Detailed publication characteristics in clinical chemistry, 2005–2022.

Characteristics (N = 30,268)	**Overall**, N = 30,268^a^	First Author's Gender			p-value
		**Female**, N = 12,861^a^	**Male**, N = 16,672^a^	**Unknown**, N = 735^a^	
Last Author's Gender					<0.001^d^
Female	8175/30,268 (27 %)	4591/12,861 (36 %)	3411/16,672 (20 %)	173/735 (24 %)	
Male	20,389/30,268 (67 %)	7521/12,861 (58 %)	12,431/16,672 (75 %)	437/735 (59 %)	
Unknown	1704/30,268 (5.6 %)	749/12,861 (5.8 %)	830/16,672 (5.0 %)	125/735 (17 %)	
Number of Authors	5.00 (3.00, 8.00)	6.00 (4.00, 8.00)	5.00 (3.00, 8.00)	6.00 (3.00, 8.00)	<0.001^e^
Publication Year	2016 (2011, 2019)	2016 (2012, 2020)	2015 (2011, 2019)	2016 (2012, 2019)	<0.001^c^
Usage Count (Since 2013)	4.00 (2.00, 9.00)	4.00 (2.00, 9.00)	4.00 (2.00, 9.00)	4.00 (2.00, 10.00)	0.63^e^
Times Cited in Web of Science	9.00 (3.00, 25.00)	8.00 (2.00, 22.00)	10.00 (3.00, 26.00)	9.00 (2.00, 24.00)	<0.001^e^
Total Times Cited Count	9.00 (3.00, 26.00)	9.00 (2.00, 23.00)	10.00 (3.00, 28.00)	9.00 (2.00, 25.00)	<0.001^e^
Impact Factor 2022	4.00 (2.00, 6.00)	3.00 (2.00, 6.00)	4.00 (2.00, 7.00)	3.00 (2.00, 6.00)	<0.001^e^
Missing	665	277	378	10	
Title Word Count	14.00 (11.00, 18.00)	15.00 (11.00, 18.00)	14.00 (10.00, 18.00)	14.00 (11.00, 18.00)	<0.001^e^
Abstract is provided	25,771/30,268 (85 %)	11,431/12,861 (89 %)	13,701/16,672 (82 %)	639/735 (87 %)	<0.001^d^
Author Keywords are provided	20,548/30,268 (68 %)	9258/12,861 (72 %)	10,808/16,672 (65 %)	482/735 (66 %)	<0.001^d^
Document Type					<0.001^d^
Article	22,385/22,385 (100 %)	10,028/22,385 (45 %)	11,779/22,385 (53 %)	578/22,385 (2.6 %)	
Editorial	2296/2296 (100 %)	612/2296 (27 %)	1640/2296 (71 %)	44/2296 (1.9 %)	
Letter	1872/1872 (100 %)	684/1872 (37 %)	1153/1872 (62 %)	35/1872 (1.9 %)	
Other	279/279 (100 %)	119/279 (43 %)	150/279 (54 %)	10/279 (3.6 %)	
Review	3436/3436 (100 %)	1418/3436 (41 %)	1950/3436 (57 %)	68/3436 (2.0 %)	
International Collaboration	6500/6500 (100 %)	2594/6500 (40 %)	3730/6500 (57 %)	176/6500 (2.7 %)	<0.001^d^
Region according to the UN					<0.001^d^
Eastern Europe	1401/1401 (100 %)	837/1401 (60 %)	544/1401 (39 %)	20/1401 (1.4 %)	
Northern America	11,287/11,287 (100 %)	4301/11,287 (38 %)	6663/11,287 (59 %)	323/11,287 (2.9 %)	
Northern Europe	3709/3709 (100 %)	1473/3709 (40 %)	2144/3709 (58 %)	92/3709 (2.5 %)	
Southern Europe	5395/5395 (100 %)	2761/5395 (51 %)	2603/5395 (48 %)	31/5395 (0.6 %)	
Western Europe	7015/7015 (100 %)	2837/7015 (40 %)	3965/7015 (57 %)	213/7015 (3.0 %)	
Western or Central Asia	1461/1461 (100 %)	652/1461 (45 %)	753/1461 (52 %)	56/1461 (3.8 %)	

2 Bonferroni correction for multiple testing.

a Median (IQR) or Frequency (%).

c One-way ANOVA.

d Pearson's Chi-squared test.

e Kruskal-Wallis rank sum test 3Geographical Differences.

There were also geographical differences in the proportion of female first authors (q < 0.001), with Southern Europe having an almost balanced proportion of 51 % female first authors and Eastern Europe having the highest proportion at 60 % (Table 1, Fig. 2f, Supplemental Fig. 1).

## 5 Discussion

1 Introduction

While diversity has been shown to increase productivity [4,5] as well as broaden research interests and priorities [2,[6], [7], [8]], women remain underrepresented in many scientific disciplines [9,10].

2. Gender distribution of first authors

In this context, the observed female first authorship rate of 42 % signifies an encouraging trend towards gender parity in authorship within Clinical Chemistry and Laboratory Medicine. This trend is corroborated by Annesley, who demonstrated that female first authors contributed to 44–60 % of invited research in the journal Clinical Chemistry from 2018 to 2020, and for 33–59 % in the Journal of Applied Laboratory Medicine from 2017 to 2020 [27]. In regard to original research articles, females accounted for 43 % of first authors in the Journal of Applied Laboratory Medicine and 49 % in the journal Clinical Chemistry during the period of 2018–2019 [28]. Notably, this represents a stark contrast to the 29 % representation of female first authors in Clinical Chemistry during 1998–1999 [28], further underscoring the increase in female first authorships over time identified in this study.

Improved working environments, the reduction of stereotypes and more representation of women in leadership positions, editorial boards and professional societies [29,30] may have contributed to this growing inclusion of women in relevant Laboratory Medicine and Clinical Chemistry authorship positions.

Similar positive trends were found in chemistry (35.04 %), pharmacology (45.68 %), and medicine (41.97 %) in a large-scale analysis from 2002 to 2018 [10]. In Europe, 40 % of clinical chemists have a degree in medicine, 27 % in chemistry and 21 % in pharmacology [17]. Thus, the similarity between the representation of female first authors in medicine and Clinical Chemistry may be explained by the high proportion of clinical chemists with a medical background. This pattern extends to the composition of the workforce, where women constituted 53 % and 46 % of the medical profession in Europe and America, respectively, in 2019 [31]. In comparison, women represented merely 35 % of the workforce in STEM within the United States in 2021 [32], and 32 % of graduates in member countries of the Organisation for Economic Co-operation and Development in 2019 [33].

3. Gender distribution regarding country affiliation

After all, there is evidence to suggest that the gender composition of fields predicts the distribution of first authorships, with male-dominated fields showing slower improving gender ratios [10]. This could also explain geographical differences in the gender distribution of first authors, as the composition of a field's workforce is country specific. Thus, the high proportion of female first authors in Clinical Chemistry and Laboratory Medicine as well as across other disciplines in Eastern Europe [10,16] echoes the high proportion of female scientists (49 %) [34], which the literature attributes to historical and cultural gender roles as well as institutional conditions [10,16].

In contrast, only Clinical Chemistry and Laboratory Medicine seem to have a high proportion of female first authors in Southern Europe according to the literature [10,16,35]. Thus, the gender distribution of female first authors may not be determined by national or disciplinary conditions alone, but by their interaction. In this context, there appears to be a particular emphasis on Clinical Chemistry and Laboratory Medicine in Southern and Eastern Europe, reflecting the density of clinical chemists in relation to the population. Moreover, responsibilities, and therefore career opportunities, are less likely to depend solely on a particular academic qualification in these regions [21]. As well as encouraging interdisciplinary collaboration and friendly competition between researchers of all gender, these considerations might also attract female scientists whose career paths would otherwise have taken them to another country or field.

4. Gender distribution in last authorships

Despite these positive trends, only one in four last authors in Laboratory Medicine and Clinical Chemistry was female, mirroring other disciplines [10]. This transdisciplinary phenomenon is metaphorically termed the leaky pipeline, which highlights the progressive loss of women from STEM fields as they advance in their careers [36]. Whithin academia, this phenomenon is often attributed to workplace inequality, institutional bias, less funding as well as fewer authorship credits for equal scientific contribution [37]. The obstacles for women begin as early as the hiring phase, characterized by imbalances in candidate profiles, imperfect recommendation systems, and biased evaluations [38]. Moreover, female researchers often receive less recognition from professional societies, manifested through fewer awards [39] and mentions in newsletters [40]. This diminished recognition extends to receiving fewer invitations to speak at scientific conferences, potentially limiting the dissemination and citation of their work [41].

The journey continues with the peer review [14,42,43] and editorial processes [10,44,45], where the contributions of women are more likely to face bias, further exacerbating the disparities. The literature also describes bias in the allocation of awards [46] and research grants [47], where not only are women less frequently recipients, but the awards they do receive generally carry lower monetary value [46].

These findings resonate with the research conducted by Hofstra et al., which highlight the systematic undervaluing and discounting of contributions from underrepresented groups, despite their often more innovative nature [48]. Beyond these implicit and explicit bias [30,49], women within the Association for Diagnostics and Laboratory Medicine also articulate difficulties in reconciling professional responsibilities with familial duties [30]. This struggle illustrates a wider societal challenge in which expectations of women's roles act as a pervasive barrier.

However, the proportion of female last authors will likely rise, as today's first authors are positioned to become tomorrow's last authors [50]. The implications of the observed gender concordance between first and last authors may even reinforce this. This gender concordance is also reported in other disciplines [51,52], where it is associated with higher publication and citation rates [51]. Similar conflict management in authorship disputes [13], as well as the impact of female representation on work climate [53] and workplace equality [54] may foster these constellations. If confirmed, these gender-role rather than gender-dependent factors may provide a basis for promoting diversity in research groups.

5. Gender distribution in citations

The literature suggests, gender roles may also contribute to lower levels of international collaboration among female authors, resulting in their lower citation metrics across science [16]. Yet, within the domain of Clinical Chemistry and Laboratory Medicine, our findings reveal that neither international collaboration nor the gender of the first or last authors serves as a determinant of citation frequencies. Rather, alternative factors such as the recency of publication, the nature of the document, and the number of authors may account for the reduced citation rates associated with female authored publications.

In all likelihood, once women establish themselves in Clinical Chemistry, their scientific achievements will be as visible and impactful as those of their male colleagues. This hypothesis is supported by the statistically insignificant odds ratios for female last authorship with usage count since 2013, as well as impact factor. Thus, it seems plausible that female scientists might benefit from the niche potential encouraged by the multifaceted nature of this specific field [16], although further studies are needed to validate and investigate this phenomenon.

## 6 Future directions

While there is a clear trend toward equal authorship, enhancing the visibility of female authors in Clinical Chemistry and Laboratory Medicine, as well as in the wider scientific community, ensures equality. To overcome the challenges women face in academia, adopting policies that facilitate their career development is critical. For instance, women of the Association for Diagnostic and Laboratory Medicine suggest fostering a supportive environment that includes childcare facilities and the introduction of comprehensive maternity and paternity leave policies [30]. Moreover, the research by Ayala-Lopez et al. highlights additional approaches for addressing bias, advocating for deeper introspection, financial support, training as well as refresher courses on Diversity, Equity, and Inclusion [49]. Overall, to effectively advance Diversity, Equity, and Inclusion in academia, further research is needed to identify and implement effective strategies as well as policies.

## 7 Limitations

The study design and necessary methodological assumptions impose several limitations to this study. The retrospective nature of the study prohibits causal assumptions. Due to the exclusion criteria, this study's findings may not be representative of future publications or publications outside the geographical regions studied. As with any bibliometric analysis, self-citation, language barriers, and accessibility of specific journals might skew the data. While the use of a search query allows for journal independence in the construction of the bibliometric collection, it also carries the risk of identifying thematically divergent publications. Moreover, the importance of an author may be overestimated by exceptions to the usual order of authorship. Although verified algorithms were used, deviations from the expected gender name patterns could lead to misclassification of gender. Furthermore, a significant limitation of this study is its dependence on a binary gender classification system. While this methodology facilitates the identification of certain patterns, it fails to capture the comprehensive spectrum of gender identities adequately. Considering the multifaceted nature of gender, which transcends societal constructs, the retrospective categorization of authors' genders based solely on their names does not accurately reflect gender diversity. An improved strategy might include prospectively requesting authors to self-identify their gender, thereby offering a more nuanced understanding of diversity within scientific publishing. Nevertheless, the adoption of such a measure might lead to unintended repercussions, including the potential for misuse of this information and its impact on the author submission process. Given that these considerations exceed the scope of this study, future research is needed to examine the advantages and drawbacks of such methodologies. This is essential to formulate precise recommendations for enhancing research on gender distribution among authors.

## 8 Conclusion

Clinical Chemistry and Laboratory Medicine shows a promising trend towards gender balance in authorship, despite persistent challenges for female authors such as fewer citations and the 'leaky pipeline'. The strong representation of first authors from Southern Europe in Clinical Chemistry and Laboratory Medicine suggests a possible interaction between region and discipline, with possible implications for promoting equity in scientific authorship. Moreover, the observed unique balance between citations of female and male last authors in the field of Clinical Chemistry and Laboratory Medicine may provide opportunities for future research to increase the visibility of female scientists.

## Other disclosures

None.

## Ethic approval

All aspects of this research project comply with the ethical standards of the Ethics Committee of the University Hospital of Cologne, which reviewed and approved the project on February 07, 2023.

## Disclaimers

None.

## Previous presentations

None.

## Generative AI

During the preparation of this work the authors used ChatGPT (GPT-4) and DeepL for linguistic and translation purposes as well as to assist with the coding process for the statistic analysis. After using this tool/service, the authors reviewed and edited the content as needed and take full responsibility for the content of the publication.

## Data availability

The authors are unable to share the underlying dataset due to proprietary constraints and ethical restrictions imposed by the ethics committee, which prohibit the dissemination of the data.

## CRediT authorship contribution statement

**Annika Meyer:** Writing – original draft, Visualization, Software, Methodology, Investigation, Formal analysis, Data curation, Conceptualization. **Thomas Streichert:** Writing – review & editing, Validation, Supervision, Resources, Project administration, Funding acquisition, Conceptualization.

## Declaration of competing interest

The authors declare that they have no known competing financial interests or personal relationships that could have appeared to influence the work reported in this paper. The authors acknowledge support for the Article Processing Charge from the 10.13039/501100001659DFG (German Research Foundation, 491454339).

## List of abbreviations

- STEM: science, technology, engineering and mathematics
