# Supplementary material for: Towards equal representation - A bibliometric analysis of authorships in Laboratory Medicine and Clinical Chemistry from the United States, Canada, and Europe (2005–2022)
Source: Heliyon. 2024 May 16;10(10):e31411. doi: 10.1016/j.heliyon.2024.e31411 (PMC11141379; doi:10.1016/j.heliyon.2024.e31411)
Supplement: Multimedia component 1 [file mmc1.docx]

**Supplemental 1:**

In addition to the programming language R (1), we used 'rio' (2), 'tidyverse' (3, 4), 'scales' (5) and 'reshape2' (6) for data preparation. For descriptive statistics and regressions we used "gtsummary" (7, 8), "labelled" (9) and "bibliometrix" (10). For the graphical presentation of our data we used 'ggplot' (11, 12), 'ggpubr' (13), 'cowplot' (14).

**Literature**

1. Team RC. R: A Language and Environment for Statistical Computing. Vienna, Austria: R Foundation for Statistical Computing; 2022.

2. Chan C-h, Leeper TJ. rio: A Swiss-Army Knife for Data I/O. 2021 2021//.

3. Wickham H. tidyverse: Easily Install and Load the Tidyverse. 2022.

4. Wickham H, Averick M, Bryan J, Chang W, McGowan LDA, François R, et al. Welcome to the {tidyverse}. Journal of Open Source Software. 2019. p. 1686.

5. Seidel HWaD. scales: Scale Functions for Visualization. 2022.

6. Wickham H. Reshaping data with the reshape package. Journal of Statistical Software 2007;21:12.

7. Sjoberg DD, Larmarange J, Curry M, Lavery J, Whiting K, Zabor EC. gtsummary: Presentation-Ready Data Summary and Analytic Result Tables. 2022.

8. Sjoberg DD, Whiting K, Curry M, Lavery JA, Larmarange J. Reproducible Summary Tables with the gtsummary Package. {The R Journal}. 2021. p. 570-80.

9. Larmarange J. labelled: Manipulating Labelled Data. 2022.

10. Cuccurullo MAaC. bibliometrix: An R-tool for comprehensive science mapping analysis. Journal of Informetrics 2017 as doi: 10.1016/j.joi.2017.08.007.

11. Wickham H. ggplot2: Elegant Graphics for Data Analysis. Springer-Verlag New York; 2016.

12. Wickham H, Chang W, Henry L, Pedersen TL, Takahashi K, Wilke C, et al. ggplot2: Create Elegant Data Visualisations Using the Grammar of Graphics. 2022.

13. Kassambara A. ggpubr: ggplot2 Based Publication Ready Plots. 2022 2022//.

14. Wilke CO. cowplot: Streamlined Plot Theme and Plot Annotations for ggplot2. 2020.
