# Supplementary material for: Towards equal representation - A bibliometric analysis of authorships in Laboratory Medicine and Clinical Chemistry from the United States, Canada, and Europe (2005–2022)
Source: Heliyon. 2024 May 16;10(10):e31411. doi: 10.1016/j.heliyon.2024.e31411 (PMC11141379; doi:10.1016/j.heliyon.2024.e31411)
Supplement: Multimedia component 2 [file mmc2.pdf]

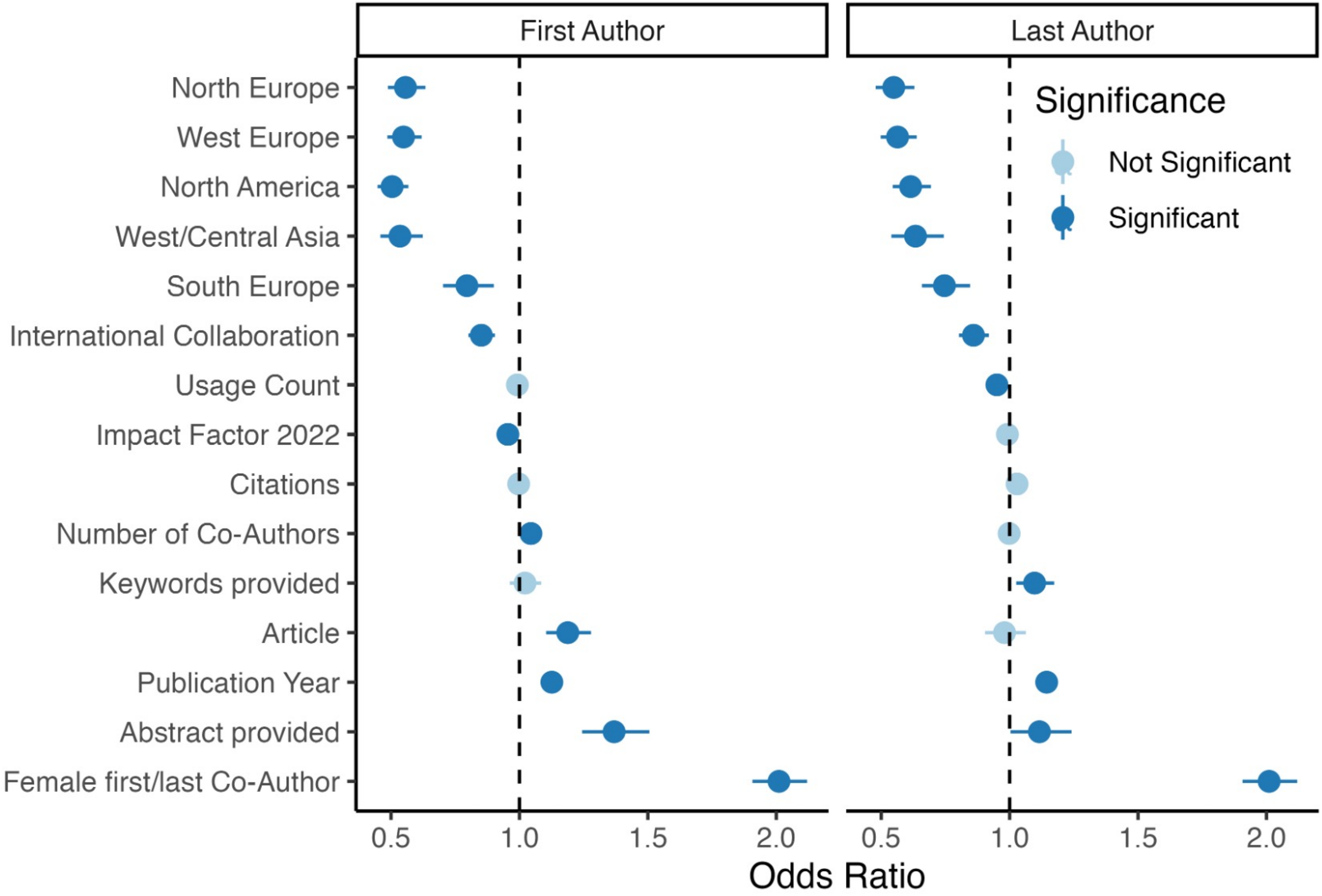

Appendix 2: Odds ratio for female first and last authorship in clinical chemistry, 2005-2022. Odds ratio is presented with interquartile range. Statistical significance is assumed at P values < 0.05. As a result of the literature search, Eastern Europe was used as a reference category for the author's affiliations.
