# Supplementary material for: Towards equal representation - A bibliometric analysis of authorships in Laboratory Medicine and Clinical Chemistry from the United States, Canada, and Europe (2005–2022)
Source: Heliyon. 2024 May 16;10(10):e31411. doi: 10.1016/j.heliyon.2024.e31411 (PMC11141379; doi:10.1016/j.heliyon.2024.e31411)
Supplement: Multimedia component 3 [file mmc3.docx]

**Supplemental Table 1: Multivariate regression of citation rates in clinical chemistry and laboratory medicine publications, 2005-2022.**

| **Characteristic** | **Beta** | **95% CI***^1^* | **p-value** |
| --- | --- | --- | --- |
| First Author's Gender |  |  |  |
| Female | — | — |  |
| Male | 0.78 | -2.2, 3.7 | 0.6 |
| Unknown | -11 | -20, -1.2 | 0.027 |
| Last Author's Gender |  |  |  |
| Female | — | — |  |
| Male | -3.0 | -6.3, 0.25 | 0.070 |
| Unknown | -5.8 | -12, 0.83 | 0.086 |
| Number of Authors | 0.71 | 0.50, 0.93 | <0.001 |
| Publication Year | -2.0 | -2.3, -1.7 | <0.001 |
| Impact Factor 2022 | 1.2 | 1.0, 1.4 | <0.001 |
| Usage Count (Since 2013) | 3.0 | 2.9, 3.0 | <0.001 |
| Abstract is provided |  |  |  |
| FALSE | — | — |  |
| TRUE | 9.7 | -1.3, 21 | 0.085 |
| Author Keywords are provided |  |  |  |
| FALSE | — | — |  |
| TRUE | -8.8 | -12, -5.1 | <0.001 |
| Document Type |  |  |  |
| Article | — | — |  |
| Editorial | -5.1 | -16, 6.1 | 0.4 |
| Letter | 1.9 | -10, 14 | 0.8 |
| Other | -7.2 | -25, 11 | 0.4 |
| Review | -14 | -18, -9.0 | <0.001 |
| International Collaboration |  |  |  |
| FALSE | — | — |  |
| TRUE | 3.2 | -0.38, 6.8 | 0.080 |
| Region according to the UN |  |  |  |
| Eastern Europe | — | — |  |
| Northern America | 17 | 10, 24 | <0.001 |
| Northern Europe | 13 | 5.5, 21 | <0.001 |
| Southern Europe | 13 | 5.2, 20 | <0.001 |
| Western Europe | 11 | 3.3, 18 | 0.004 |
| Western or Central Asia | 12 | 2.8, 21 | 0.011 |
|  | | | |
| *^1^* CI = Confidence Interval | | | |
